# Being open: our policy on source code

**DOI:** 10.1186/s13059-016-1040-y

**Published:** 2016-08-12

**Authors:** Rafal T. Marszalek, Louisa Flintoft

**Affiliations:** Genome Biology, BioMed Central, 236 Gray’s Inn Road, London, WC1X 8HB UK

## ᅟ

Fifteen years ago *Genome Biology* published its first Software article [1]. It described a suite of programs for the analysis of microarray data. The tool was deposited on the authors’ website (now defunct) and was “copyrighted against commercial gain”. The conditions of deposition and release reflected the understanding of and the reservations about open source that the scientific community had at the beginning of this century.

Many readers will be aware that this would not stand today: the times have changed, the field has matured, and the definition of open science has crystallized. Many researchers now acknowledge that openness and transparency foster better, reproducible research. A Comment published in our journal last year argued that all research should be carried out openly, so that what we today call ‘open science’ should just become ‘science’ [2]. One recent study also showed that, despite common misconceptions, open science can actually aid researchers’ careers [3].

With all the benefits that open science brings, our authors and readers could not have been surprised in the past several years to see us require that any source code published as a part of an article is deposited in a recognized public source code repository [4]. Since the beginning of 2016 we have also started requesting that the authors of Method and Software articles describing new computational tools archive the version used in the manuscript in a DOI-assigning repository, such as Figshare [5] or Zenodo [6]. This way the researchers trying to reproduce analyses described in the article can use the exactly same code as the authors - inoculating those analyses against the inevitable evolution of code.

We consider source code availability and accessibility to be important to ensure that computational analyses can be easily repeated and research shown to be reproducible. It is, however, no less critical that the source code can be utilized by others to further their own research: that other people can use it, and develop it, and build on it (and cite it [7]), and create new and wonderful tools that the authors of the original may not have even dreamed of. And an important factor affecting the ability to do this is the source code license.

This is why *Genome Biology* requires that the source code published as a part of an article is released under a license complying with an Open Source Definition, as defined by the Open Source Initiative [8]. This means that the source code can be used, modified, and distributed by anyone (importantly, the definition assures no discrimination against fields of endeavor; for more see Box). We believe that, together with a robust source code archiving strategy, employing open source licenses serves to preserve the source code long-term — through its re-use, re-purposing, and further evolution. And thus it really helps it become a foundation of research not yet imagined.

## Box 1. Open Source Definition and “fields of endeavour”

One of the open source code criteria, according to the Open Source Definition, is no discrimination against fields of endeavor. What this means in practice is that tools released under OSI-compliant licenses cannot use the “for non-commercial use only” disclaimers. A common worry [9] is that this affects whether the tool can be commercialized:

***Do open source licenses stop me from commercializing my code?***

No – no matter which open source license is used.

***Do open source licenses mean that others can commercialize my code?***

By definition, the open source code can be commercialized by anyone: the code’s author, their institution, an independent commercial entity, a private person. Literally anyone. This is independent of the exact OSI-compliant license used.

***How do I choose a license?***

The good folks of Github came to the rescue and created the ‘Choose an open source license’ service [10] for this. You can also browse for an appropriate license on the Open Source Initiative website [8]. Although some users believe that open source code should never be released under licenses that are “copyleft” [11] (these are the licenses which require that any derivative of the code is released under the same license), an equally good argument can be made [12] for GPL license-compatible licenses (which often are “copyleft”). While the use of permissive licenses that are not “copyleft” is more in the spirit of open science, *Genome Biology* currently accepts all OSI-compliant licenses.

***How do I go about licensing my source code from the legal point of view?***

Only the owner of the intellectual property rights to the source code can license it. While *Genome Biology* requires that the source code is released under an OSI-compliant license, it is the authors’ responsibility to ensure that they are within their rights to use such a license in the first place. Morin and colleagues provide a handy guide for the authors on what to do before you release your code under any license [13].
